# Children as sentinels of tuberculosis transmission: disease mapping of programmatic data

**DOI:** 10.1186/s12916-020-01702-x

**Published:** 2020-09-02

**Authors:** Kenneth S. Gunasekera, Jon Zelner, Mercedes C. Becerra, Carmen Contreras, Molly F. Franke, Leonid Lecca, Megan B. Murray, Joshua L. Warren, Ted Cohen

**Affiliations:** 1 Department of Epidemiology of Microbial Diseases, Yale School of Public Health, 60 College Street, New Haven, CT 06520 USA; 2 Department of Epidemiology, University of Michigan School of Public Health, 267 SPH Tower, 1415 Washington Heights, Ann Arbor, MI 48109 USA; 3 Department of Global Health and Social Medicine, Harvard Medical School, 641 Huntington Avenue, Boston, MA 02115 USA; 4 Socios En Salud, Lima, Peru; 5 Department of Biostatistics, Yale School of Public Health, 60 College Street, New Haven, CT 06520 USA

**Keywords:** Spatial, Hotspot, Tuberculosis, Modeling, Age-structure, Pediatric, Population surveillance/*methods, Tuberculosis/epidemiology/*prevention & control/transmission

## Abstract

**Background:**

Identifying hotspots of tuberculosis transmission can inform spatially targeted active case-finding interventions. While national tuberculosis programs maintain notification registers which represent a potential source of data to investigate transmission patterns, high local tuberculosis incidence may not provide a reliable signal for transmission because the population distribution of covariates affecting susceptibility and disease progression may confound the relationship between tuberculosis incidence and transmission. Child cases of tuberculosis and other endemic infectious disease have been observed to provide a signal of their transmission intensity. We assessed whether local overrepresentation of child cases in tuberculosis notification data corresponds to areas where recent transmission events are concentrated.

**Methods:**

We visualized spatial clustering of children < 5 years old notified to Peru’s National Tuberculosis Program from two districts of Lima, Peru, from 2005 to 2007 using a log-Gaussian Cox process to model the intensity of the point-referenced child cases. To identify where clustering of child cases was more extreme than expected by chance alone, we mapped all cases from the notification data onto a grid and used a hierarchical Bayesian spatial model to identify grid cells where the proportion of cases among children < 5 years old is greater than expected. Modeling the proportion of child cases allowed us to use the spatial distribution of adult cases to control for unobserved factors that may explain the spatial variability in the distribution of child cases. We compare where young children are overrepresented in case notification data to areas identified as transmission hotspots using molecular epidemiological methods during a prospective study of tuberculosis transmission conducted from 2009 to 2012 in the same setting.

**Results:**

Areas in which childhood tuberculosis cases are overrepresented align with areas of spatial concentration of transmission revealed by molecular epidemiologic methods.

**Conclusions:**

Age-disaggregated notification data can be used to identify hotspots of tuberculosis transmission and suggest local force of infection, providing an easily accessible source of data to target active case-finding intervention.

## Background

The End TB Strategy’s ambitious goals to reduce tuberculosis incidence require new interventions to interrupt transmission [1]. This has led to a renewed interest in active case-finding strategies, in which risk groups are screened to identify infectious individuals before they present to care [2, 3]. Because untargeted community-based active case-finding has not consistently demonstrated population-level benefits [4–7], there has been interest in new practical approaches to focus case-finding to population groups among whom risk is concentrated. One such approach is to target active case-finding to hotspots, areas in which transmission is most intense [8]. While evidence supporting the impact of targeting screening in hotspots is currently limited [9], mathematical modeling suggests that such targeting can produce substantial population-wide reductions in transmission [10, 11].

Conclusive evidence of hotspot transmission typically relies on access to detailed spatial and pathogen genetic data [12–14]. While spatial information is often available in public health reporting systems (e.g., home location), in high-transmission/lower-income settings, resources for genetic sequencing of pathogens are typically only available in research studies. Thus, methods to robustly identify hotspots from routine reporting data would be valuable [15]. However, given that high local rates of tuberculosis notifications may reflect spatially aggregated risk for progression of infection, migration of individuals infected with tuberculosis into the area [16], or spatial heterogeneity in diagnostic capacity [17], finding new ways to probe routine surveillance data to find evidence of local transmission is a priority.

Spatial differences in the age distribution of tuberculosis cases in a single city may provide a signal for local transmission intensity [18]. In locations where disease transmission is more intense, cases are systematically younger than in locations where disease transmission is less intense [19]. We aimed to test this previously posited, but to our knowledge yet untested, idea that areas where children are overrepresented in tuberculosis case notification data are areas where recent transmission events are concentrated. We tested this hypothesis using case notification data from Lima, Peru, where we were able to compare our inference to a prospective molecular epidemiology study conducted in the same setting several years later [20, 21]. This comparison provided an opportunity to examine whether routinely collected tuberculosis notification data can be used to identify transmission hotspots.

## Methods

### Study setting and population

We examined data from all tuberculosis cases notified to Peru’s National Tuberculosis Program from two of Lima’s four health districts, Lima Ciudad and contiguous catchment areas of Lima Este, between January 1, 2005 and December 31, 2007. Patient demographic and clinical information was available within the notification data as well as household address, which was identified on high-resolution maps created using Google Earth. Additional details of the study design and mapping procedures have been described previously [22, 23].

Our interest was in identifying areas in which young children were overrepresented in these routinely collected notification data from 2005 to 2007 and whether they correlated with areas identified as transmission hotspots during a prospective study of tuberculosis transmission conducted from 2009 to 2012 [21]. The latter study included molecular epidemiological characterization of culture-positive cases of drug-susceptible and drug-resistant tuberculosis from adults older than 15 years using 24-loci mycobacterial interspersed repetitive units-variable-number tandem repeats (MIRU-VNTR). Spatial aggregation of *Mycobacterium tuberculosis* (*M.tb*) strains identified by MIRU-VNTR genotype was presumed to indicate transmission.

### Data visualization and modeling

We visualized spatial clustering of child cases < 5 years old in the notification data using a log-Gaussian Cox process (LGCP) to model the intensity function driving the point process describing the distribution of child cases. We used the *lgcp* package and defined the Gaussian process with an exponential covariance function and weakly informative priors on all model parameters (details provided in Additional file 1: Supplementary Information) [24]. All data visualization and analysis were performed using R 4.0.1.

Next, we aimed to determine if the clustering of child cases observed in the exploratory maps was more extreme than would be expected by chance alone. Point-level census and covariate data that may explain spatial variability in the distribution of child cases through effect on overall risk were not available for this analysis. Due to the large number of unique spatial locations observed in the data (10,198) and the well-known difficulties associated with using a Gaussian process to analyze point-referenced spatial data when the sample size is large [25], we opted for a method that approximates the point-referenced model while offering computational improvements [26]. Specifically, we overlaid a grid on the convex hull of the case notification data and modeled the proportion of reported tuberculosis cases that occurred among children in each grid cell using a hierarchical Bayesian spatial modeling framework. We chose the grid cell sizes to be small in order to ensure that the risk within each grid cell was homogeneous. We considered multiple sizes in subsequent sensitivity analyses. As the size of the grid cells gets smaller, our approximation to the point-referenced geostatistical model improves.

By modeling the proportion of the tuberculosis cases that were children (as opposed to simply modeling the number of child cases), we used the distribution of adult cases to control for unobserved factors that may explain the spatial variability in the distribution of child cases. Under this modeling framework, we expect that the local proportion of child cases will be higher than the expected proportion of child cases over the entire study area in areas where there is local transmission. The hierarchical model structure allows us to identify where this occurs and allows us to describe the certainty with which the proportion is higher.

To do this, we use a logistic regression framework to model the grid cell-specific proportions such that:

$$ {Y}_i\mid {\theta}_i\sim \mathrm{Binomial}\left({n}_i,{\theta}_i\right),i=1,\dots, m $$

$$ \ln \left(\frac{\theta_i}{1-{\theta}_i}\right)=\mu +{\phi}_i $$

where *Y*_*i*_ is the number of child cases observed in grid cell *i*, *n*_*i*_ is the total number of child and adult cases in the grid cell, *m* is the total number of grid cells, and *θ*_*i*_ represents the proportion of the total cases in the grid cell that are due to children. We define child cases as those < 5 years old and adult cases as those > 15 years old to clearly separate recent infection among young children from more distant infection among adults (expecting that cases among older children and young adults between ages 5 and 15 represent a mix of recent infection and infection that happened earlier in their lives). We model these proportions on the logit scale as a function of an overall mean, *μ* (fixed effect), and a grid cell-specific deviation from that mean, *ϕ*_*i*_ (random effect).

We anticipate that the proportion of child cases in grid cells that are close together may be similar. To account for this potential spatial correlation and to obtain spatially smoothed risk estimates, we estimated the *ϕ*_*i*_ parameters using a conditional autoregressive (CAR) model such that:

$$ {\phi}_i\mid {\boldsymbol{\phi}}_{-i},{\tau}^2,\rho \sim \mathrm{N}\left(\frac{\rho {\Sigma}_{j=1}^n{w}_{ij}{\phi}_j}{\rho {\Sigma}_{j=1}^n{w}_{ij}+1-\rho },\frac{\tau^2}{\rho {\Sigma}_{j=1}^n{w}_{ij}+1-\rho}\right) $$

where ***ϕ***_−*i*_ is the vector of parameters excluding *ϕ*_*i*_, *w*_*ij*_ is equal to one if grid cells *i* and *j* share a common border or point and is equal to zero otherwise, *τ*^2^ describes the variability in the *ϕ*_*i*_ parameters, and *ρ* ∈ (0, 1) describes their strength of spatial correlation. As a result, this model is flexible enough to accommodate a wide range of spatial patterns as well as the possibility that there is no spatial variability in the proportion of child cases (i.e., *τ*^2^ near zero indicates that all *ϕ*_*i*_ are near zero). Additionally, examining the posterior distributions of *ϕ*_*i*_ allows us to determine if the grid cell proportion differs substantially from the overall mean.

We selected weakly informative prior distributions for all model parameters and used the *CAR.Leroux* function in the *CARBayes* package to obtain posterior samples for all parameters [27]. Details are provided in Additional file 1: Supplementary Information [28]. Using the posterior samples from each *ϕ*_*i*_, we estimate the posterior probability that *ϕ*_*i*_ is larger than zero, which would suggest recent transmission based on our hypothesis.

## Results

### Analysis of notification data

**Fig. 1:**
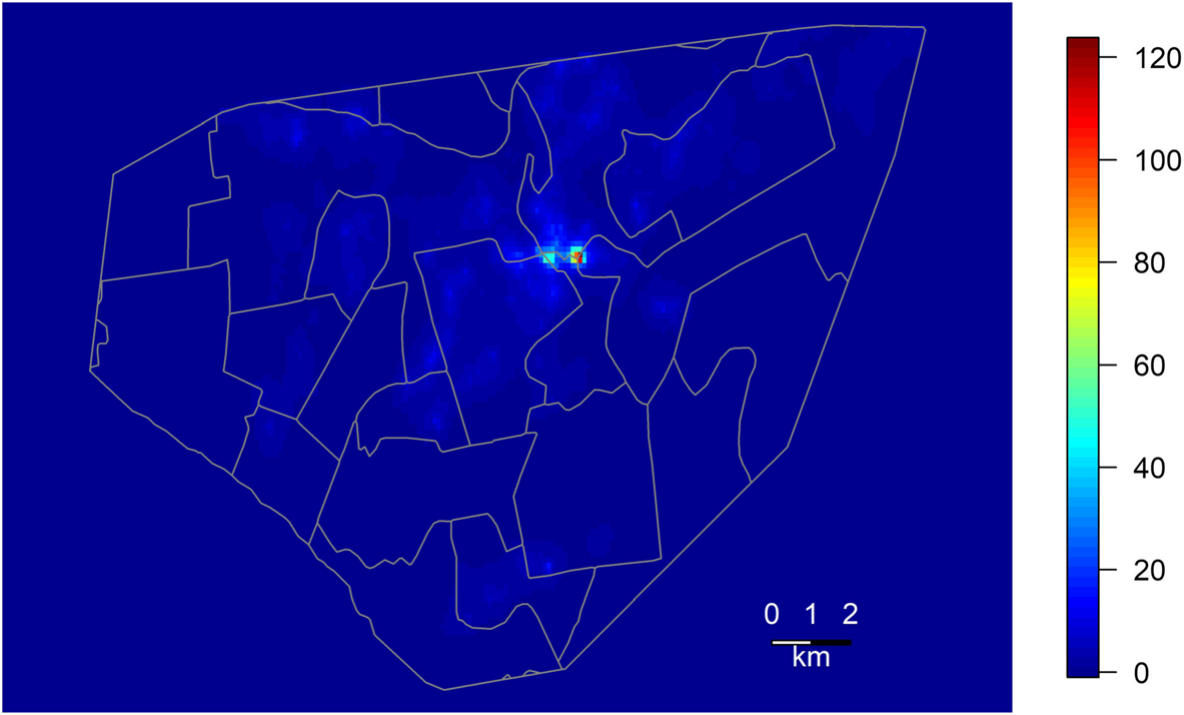
Disease mapping of young children in Peru’s National Tuberculosis Program data. Log-Gaussian Cox process modeled intensity of the cases of tuberculosis among children < 5 years old notified to the Peru’s National Tuberculosis Program within two of Lima’s four health districts, Lima Ciudad and contiguous catchment areas of Lima Este, between January 1, 2005 and December 31, 2007

Of the total 11,711 notified tuberculosis cases over the study period, there were 332 children < 5 years old, and 10,352 adults > 15 years old. The LGCP modeled intensity of the cases among children < 5 years old is given in Fig. 1.

**Fig. 2:**
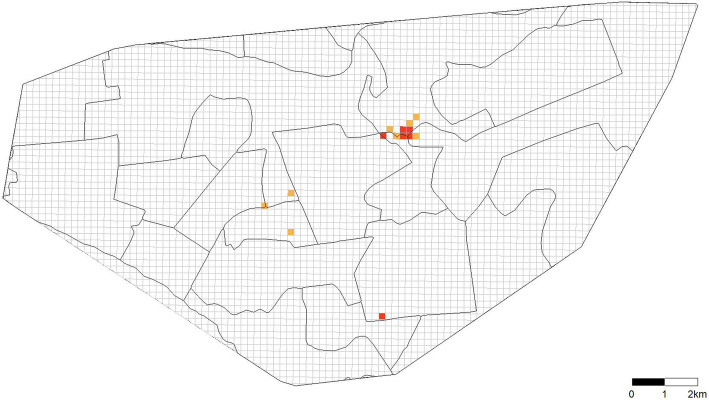
Identifying areas with local overrepresentation of young children in tuberculosis notification data. Hierarchical Bayesian spatial model fit to the child cases < 5 years old and adult cases > 15 years old in the notification data aggregated into 200 m × 200 m grid cells overlaid on the convex hull of the data. The model suggested six grid cells (red) in which > 95% of the posterior distribution of the random effect terms were above zero, and an additional eight grid cells (orange) in which > 90% of the posterior distribution was above zero. The proportion of child cases in these grid cells is greater than expected over the study region, suggesting recent tuberculosis transmission based on our hypothesis

We fit the hierarchical Bayesian spatial model to the case notification data collected from 2005 to 2007 aggregated into a 200 m × 200 m grid within the convex hull of the data. The model suggested six grid cells in which > 95% of the posterior distribution of the random effect terms were above zero and an additional eight grid cells in which > 90% of the posterior distribution was above zero (Fig. 2). Examination of the posterior estimate of the spatial correlation parameter, *ρ*, suggested that the excess variability observed in the data was spatially structured (posterior mean 0.75, 95% credible interval 0.24–0.98). Posterior summaries of the remaining parameters are provided in Additional file 1: Table S1.

### Comparison to prospective molecular epidemiological study

**Fig. 3:**
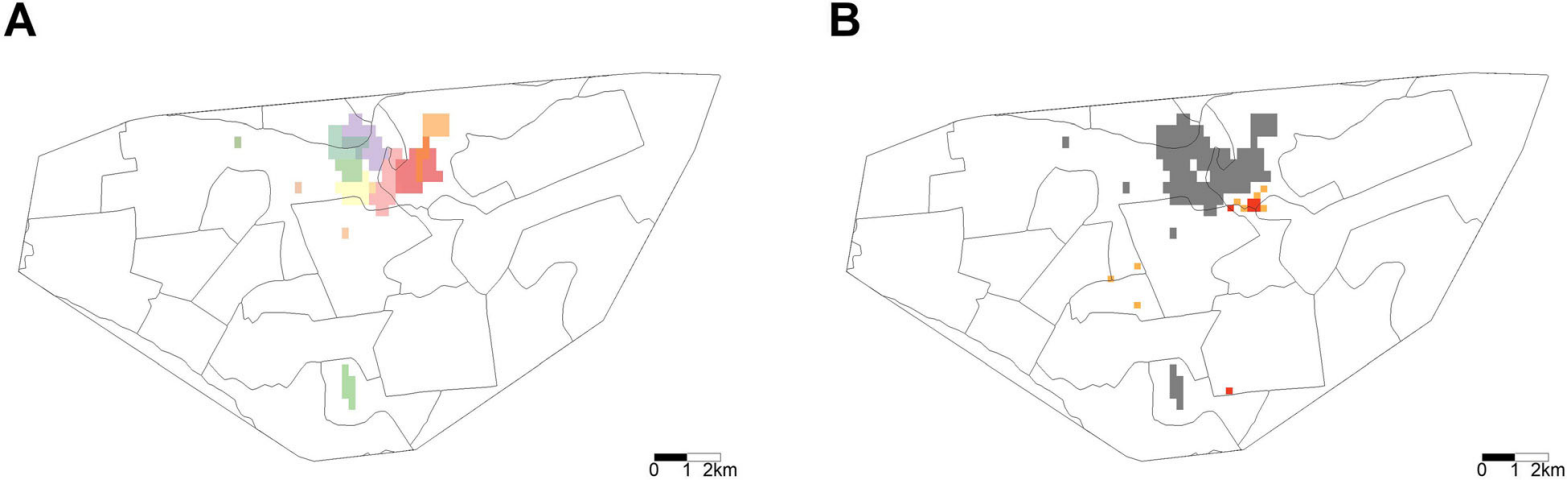
Comparing tuberculosis transmission inference of hotspots of active transmission. **a** Reproduced with permission from Zelner et al. demonstrating regions (shaded) identified as tuberculosis transmission hotspots. Different color shading denotes clusters of different drug-sensitive and drug-resistant strains identified by MIRU-VNTR genotype. **b** A grayscale reproduction of this figure is overlaid on the modeled 200 m × 200 m grid from Fig. 2. We highlight those grid cells in red and orange, where the modeled proportion of child cases < 5 years old is greater than expected, to demonstrate the proximity between areas with higher local childhood tuberculosis notification and areas with conclusive evidence of transmission. MIRU-VNTR, 24-loci mycobacterial interspersed repetitive units-variable-number tandem repeats

**Fig. 4:**
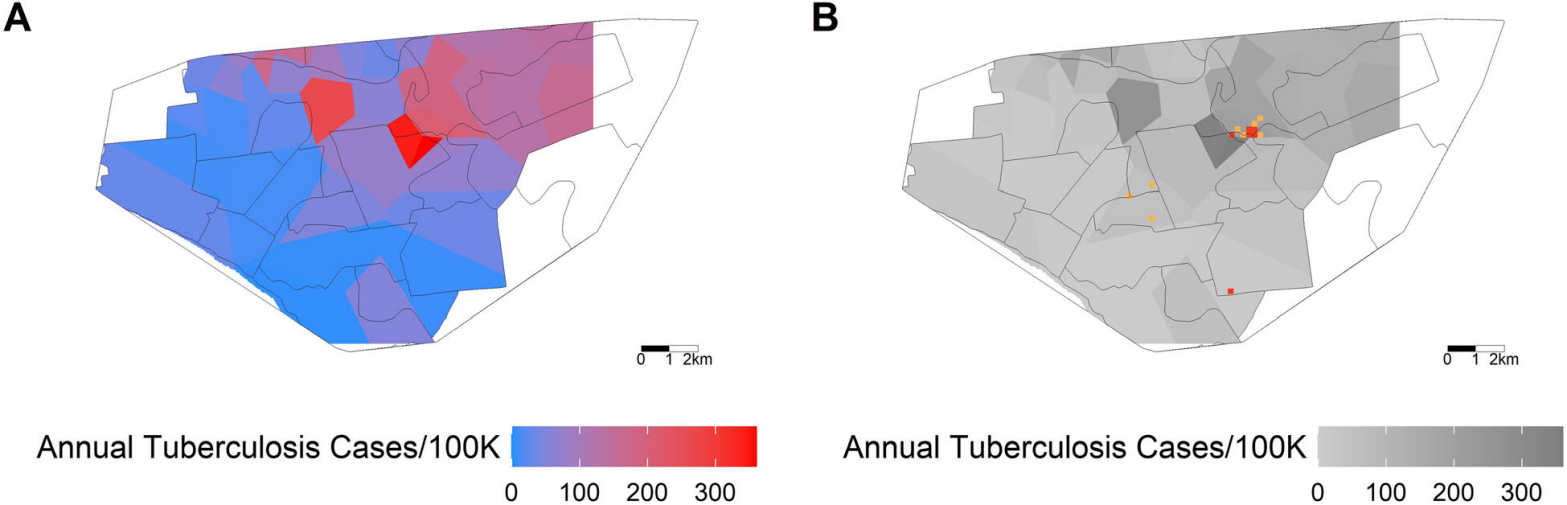
Comparing per capita tuberculosis incidence to putative hotspots. **a** Figure reproduced with permission from Zelner et al. demonstrating the spatial variation in annual per-100 thousand incidence of drug-sensitive and drug-resistant tuberculosis by healthcare catchment area. **b** A grayscale reproduction is overlaid on the 200 m × 200 m grid from Fig. 2 to demonstrate the proximity between the colored grid cells, where the modeled proportion of child cases < 5 years old is greater than expected, and an area of high local incidence of tuberculosis

Figure 3a, reproduced with permission from Zelner et al., shows areas in which there was statistically significant spatial aggregation of specific *M.tb* MIRU-VNTR genotypes, consistent with localized transmission of these strain types [21]. In Fig. 3b, we overlay the grid from Fig. 2 to demonstrate the proximity between areas where children < 5 years old are overrepresented in case notification data and areas where specific strains are concentrated. In Additional file 1: Figs. S1-S2, we show that these findings are insensitive to assumed grid cell size and age cutoffs for the definitions of young child and adult cases. Figure 4a, also reproduced with permission from Zelner et al., shows the spatial variation in annual per capita incidence of tuberculosis by healthcare catchment area [21]. We similarly overlay the grid from Fig. 2 to create Fig. 4b to demonstrate the proximity between areas where child cases are overrepresented and high local incidence.

## Discussion

In this paper, we evaluated whether routinely collected, age-disaggregated notification data can be used to identify hotspots of spatially concentrated tuberculosis transmission. Our analysis, based on routine data collected from 2005 to 2007, pinpointed a region where child cases of tuberculosis were overrepresented relative to the number of adult cases in the area. This region was previously identified as an area of high transmission using molecular genetic data from a prospective study conducted from 2009 to 2012 [21]. This concordance of transmission inference obtained using different methods and datasets supports the use of routinely collected age-disaggregated notification data to identify areas of local transmission intensity.

Child cases have been suggested as a useful signal of transmission intensity for tuberculosis as well as other infectious disease [29]. For example, a number of studies used the age prevalence of tuberculin-skin test positivity to measure risks of infection from household and community exposure [30, 31]. Previous studies have suggested that areas with high childhood tuberculosis case notification rates may correspond to areas of active transmission [32–34]; however, only one included covariates to account for potential non-transmission explanations of the spatial distribution of child cases [34]. Thus, our analysis is the first to provide molecular and epidemiological evidence to corroborate inferences of local tuberculosis transmission with attempts to control for unobserved, spatially heterogeneous, non-transmission factors that may explain the distribution of child cases (such as risk factors for progression of infection, migration of infected individuals into the area, and/or local diagnostic capacity).

Considering that both the routine notification data and the prospective molecular epidemiology study included tuberculosis cases separated by as many as 6 years, we also note that the identified hotspot appears to have been persistent over several years. This suggests that tuberculosis transmission hotspots identified from notification data may be observable for long enough periods of time to guide targeted interventions, such as spatially focused active case-finding.

It is important to note several simplifying assumptions in our analysis. Given the absence of detailed information on the distribution of covariates in the source population, we incorporated all spatial heterogeneity in the distribution of child cases into the random effect term of the model. As a result, our model necessarily attributes all spatial variability in the modeled proportions to possible recent transmission. If there are other non-transmission-related factors that impact the proportion of total cases that occurred in children, this could lead to a grid cell being incorrectly labeled as a transmission “hotspot.” However, given the consistency of our results with the previous findings that more directly measure transmission, this may not be a major issue in this work. Our hierarchical Bayesian spatial modeling approach (as well as the LGCP intensity modeling approach) is flexible enough to incorporate local covariate data as regression components. Future study should include such information when available.

Though we provide compelling evidence, we must be cautious interpreting that age-disaggregated data will always provide a reliable signal of transmission. Molecular evidence of transmission against which we compare transmission inference was only available for those > 15 years old. Thus, we are unable to biologically link childhood cases to the identified clusters of transmission. Furthermore, accurately diagnosing tuberculosis among children is difficult. While it is clear that missing child cases in notification data likely underestimate transmission, it is unclear how false positives may affect signal detection. In addition, though we demonstrate that the putative hotspot persists over time, it is not possible to assess how mobility over the time period through which all data from these two studies was collected may affect hotspot detection. It is important to note that our findings do not imply an either-or choice between genetic and age-incidence data: future analyses exploring the impact of combining granular molecular genetic data with age-incidence data in a single model could improve the predictive capacity of such models.

This methodology may be adapted to settings in which high-resolution residence data is not readily available. For example, in settings where residential geocoding is not feasible, it may be reasonable to model the proportion of child cases in the smallest recorded unit to which the household belongs (such as modeling the proportion in the neighborhood, community, and/or administrative unit).

## Conclusions

In summary, we show that age-disaggregated tuberculosis notification data may be used to investigate potential hotspots of tuberculosis transmission. This suggests that the use of models leveraging widely available data should be explored as tools for targeting case-finding and treatment efforts in high-transmission locations in the hope of maximizing the direct and indirect protective benefits of active screening approaches.

## Supplementary information

**Additional file 1 : Supplementary Information, Table S1, Figure S1-S2**. **Supplementary Information** – Log-Gaussian Cox process details and hierarchical Bayesian spatial model details. **Table S1**- Hierarchical Bayesian spatial model posterior parameter estimates. **Fig S1** – Sensitivity analysis to child and adult age cut-offs. **Fig S2** – Sensitivity analysis to grid size.

## Data Availability

Additional data are available on reasonable request to MCB and MM. All requests for data access will need to specify the planned use of data and will require approval from MCB and MM before release.

## Abbreviations

TB: Tuberculosis
MIRU-VNTR: 24-loci mycobacterial interspersed repetitive units-variable-number tandem repeats
*M.tb*: *Mycobacterium tuberculosis*
LGCP: Log-Gaussian Cox process
CAR: Conditional autoregressive
