# Supplementary material for: Children as sentinels of tuberculosis transmission: disease mapping of programmatic data
Source: BMC Med. 2020 Sep 2;18:234. doi: 10.1186/s12916-020-01702-x (PMC7466499; doi:10.1186/s12916-020-01702-x)

Additional File 1 to **Children as sentinels of tuberculosis transmission: disease mapping of programmatic data**

Kenneth S. Gunasekera, Jon Zelner, Mercedes C. Becerra, Carmen Contreras, Molly F. Franke, Leonid Lecca, Megan B. Murray, Joshua L. Warren*, Ted Cohen*

* denotes co-senior authorship

**Supplementary Information**

**Log-Gaussian Cox process details**

The log-Gaussian Cox process provides an approach to model the intensity function driving spatial point processes. Examining the fitted intensity of the cases among children <5 years old provides a robust means to identify clusters of child cases. The *lgcp* package in R models the intensity function of the spatial region that contains a regular grid with a cell width chosen to be sufficiently small to approximate continuous spatial variation. We selected a cell width of 100 m, defined the Gaussian process with an exponential covariance function, and placed weakly informative priors on all model parameters as recommended by the *lgcp* Vignette. We collected 100,000 posterior samples after discarding the first 10,000 as a burn-in period, and thinned by a factor of 90 to result in 1,000 samples from which we make posterior inference. Markov-chain Monte Carlo was performed using the Metropolis-adjusted Langevin algorithm with a target acceptance probability set to 0.574, achieved by the Andrieu and Thoms algorithm as implemented by the *lgcp* package.

**Hierarchical Bayesian spatial model details**

*Justification for small grid cell*

Small grid cells ensure that the assumption of homogenous risk within the grid cell is plausible and leads to finer scale mapping of risk across the map. Additionally, smaller grid cells allow for better approximation of a model of continuous spatial variation.

*Prior distribution specifications*

We placed weakly informative priors on the parameter describing the fixed effect term ($\mu$), the parameter describing variability in the random effect terms ($\tau^{2}$), and the parameter describing the spatial correlation parameter ($\rho$) such that:

$$\mu\sim\mathrm{Normal}(0,1000)$$

$$\tau^{2}\sim Inverse Gamma(0.01,0.01)$$

$$\rho\sim\mathrm{Uniform}(0,1)$$

*Model interpretation*

Values for $\rho$ near zero suggest near-independence of the spatial random effects while $\rho$ near one suggests a strong dependence on neighboring values (i.e., the conditional mean is an average of the neighboring values). We adopted the queen definition of neighbors because there was no data to suggest that grid cells sharing bordering points are not unrelated.

*Model convergence and posterior parameter estimation*

Model convergence was assessed using visual inspection of individual parameter traceplots and the Geweke diagnostic calculated for each parameter. Neither tool suggested obvious convergence issues (**Table S1**). In total, we collected 100,000 posterior samples after discarding the first 10,000 as a burn-in period. We further thinned the remaining samples by a factor of 10 to reduce posterior autocorrelation, resulting in 10,000 samples with which to make posterior inference.

**Sensitivity Analyses**

*Sensitivity analysis to child and adult age cut-offs*

We modeled the proportion of children of the total number of child and adult cases using different age cut-offs for both children (<2 years old, <5 years old, <15 years old) and adults (>15 years old and >25 years old). In **Fig. S1a-e**, we demonstrate that the highlighted grid cells, where the proportion of child cases is greater than expected, continue to approximate an area with molecular evidence of transmission and our findings are insensitive to the definition of children and adults.

In **Fig. S1c** where the highlighted grid cells that represent where the proportion of cases <15 years old is greater than expected do not so clearly approximate an area with molecular evidence of transmission. This is likely because young children in notification data can only have been infected during the period that they have been alive, whereas older children in notification data represent a mix of recent infection and infection that happened earlier in their lives.

*Sensitivity analysis to grid size*

We varied the size of the grid over which we aggregated the notification data to demonstrate that our proposed method is insensitive to grid size. We demonstrate this finding in **Fig. S2a-i**, where the highlighted grid cells, representing where the proportion of child cases is greater than expected, approximate the same area—irrespective of grid size—that corresponds to an area with molecular evidence of transmission.

**Table S1. Hierarchical Bayesian spatial model posterior parameter estimates**

Posterior parameter estimates and model convergence diagnostics for the hierarchical Bayesian spatial CAR model specified in the main text. This model was built using case notification data collected from 2005-2007 aggregated into a 200 m x 200 m grid using age cut-offs for children as <5 years old and adults as >15 years old.

| Model Parameter | Posterior Median (95% credible interval) | Effective Number of Independent Samples | Geweke Diagnostic Z-score |
| --- | --- | --- | --- |
| $\boldsymbol{\mu}$ | -3.6 (-3.8 – -3.5) | 475.0 | -0.2 |
| $\boldsymbol{\tau}^{\boldsymbol{2}}$ | 1.3 (0.3 – 2.6) | 243.6 | 0.1 |
| $\boldsymbol{\rho}$ | 0.7 (0.2 – 1.0) | 267.0 | -0.3 |

**Figure S1. Sensitivity analysis to child and adult age cut-offs.** Model fit using case notification data aggregated into 400 m x 400 m grid cells using different age cut-offs to define child and adult cases as follows: **(a)** child: <5 years old, adult: >15 years old (presented in the main text); **(b)** child: <2 years old, adult: >15 years old; **(c)** child: <15 years old, adult: >15 years old; **(d)** child: <5 years old, adult: >25 years old; **(e)** child: <2 years old, adult: >25 years old. We highlight those grid cells in which >90% of the modeled posterior distribution of the random effect is above zero (orange), which includes those grid cells in which >95% of the modeled posterior distribution is above zero (red).

**a)**


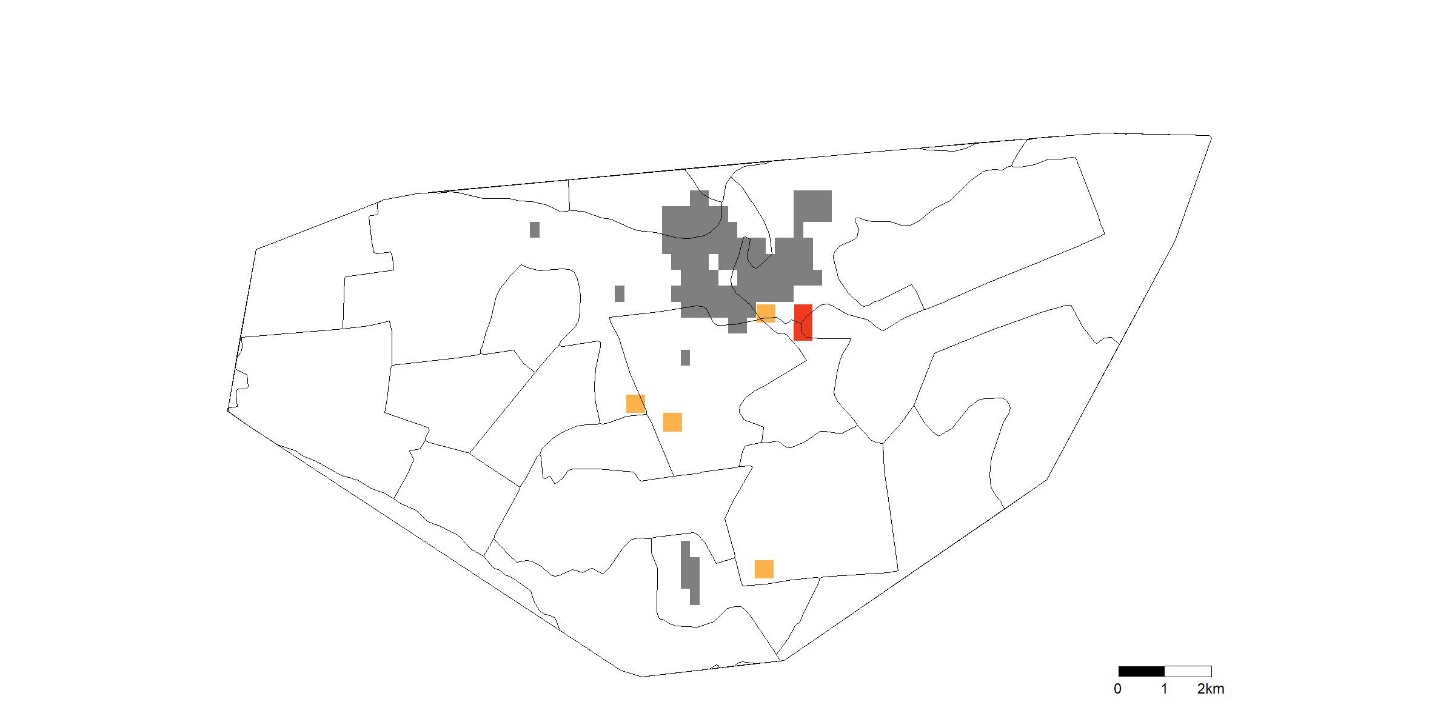


**b)**


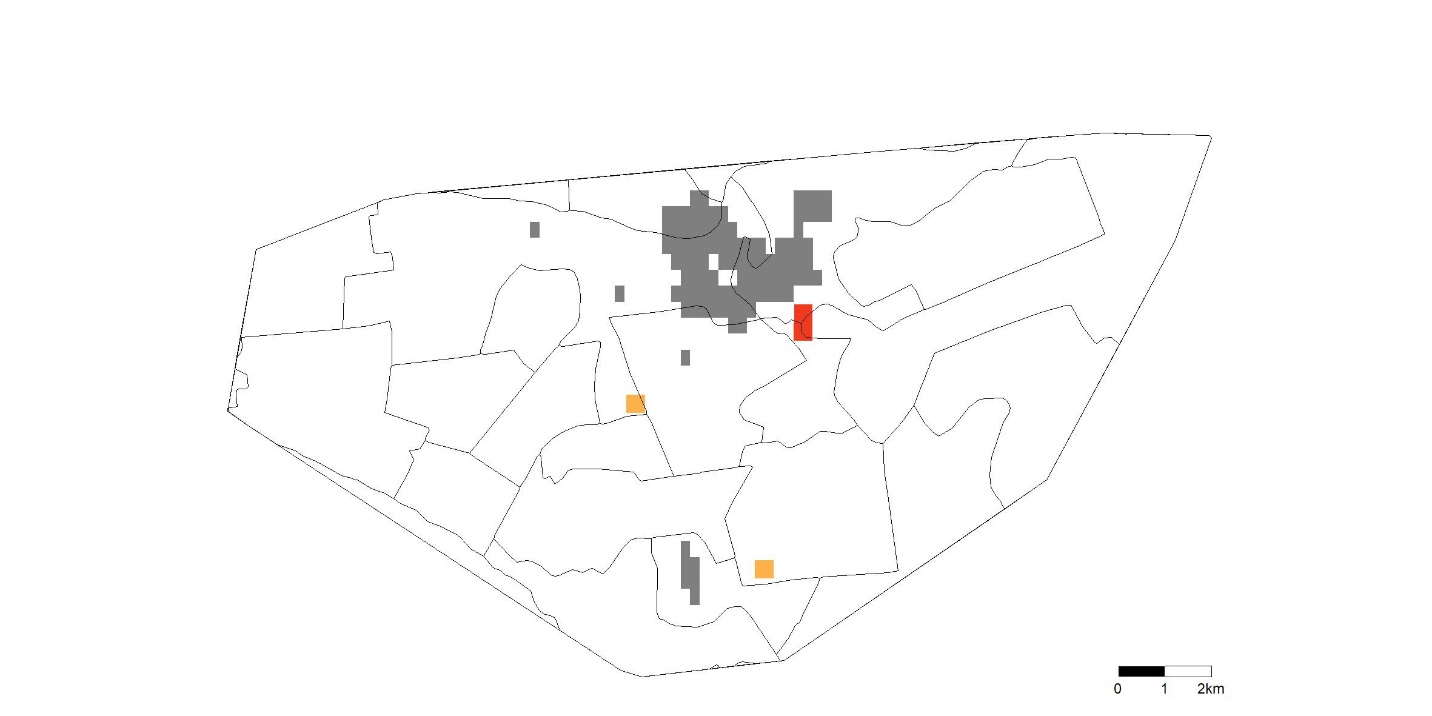


**c)**


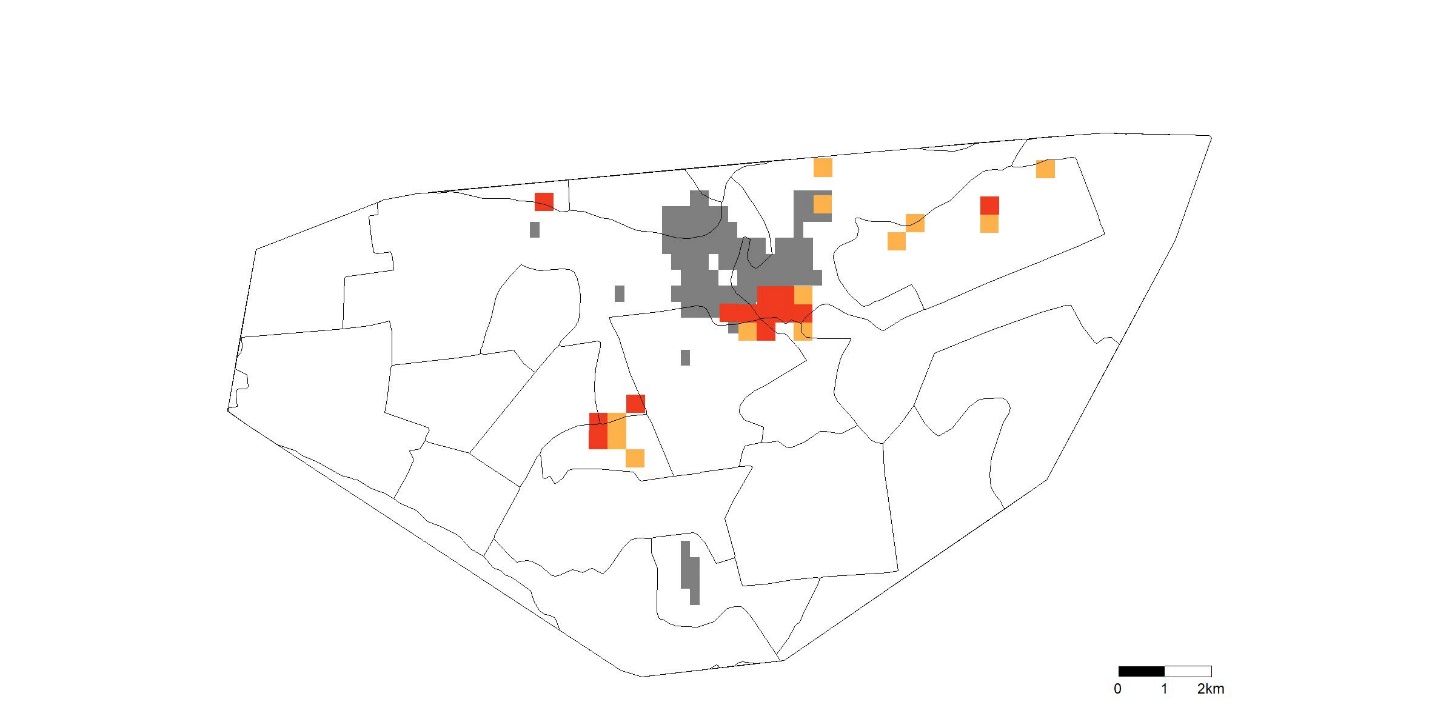


**d)**


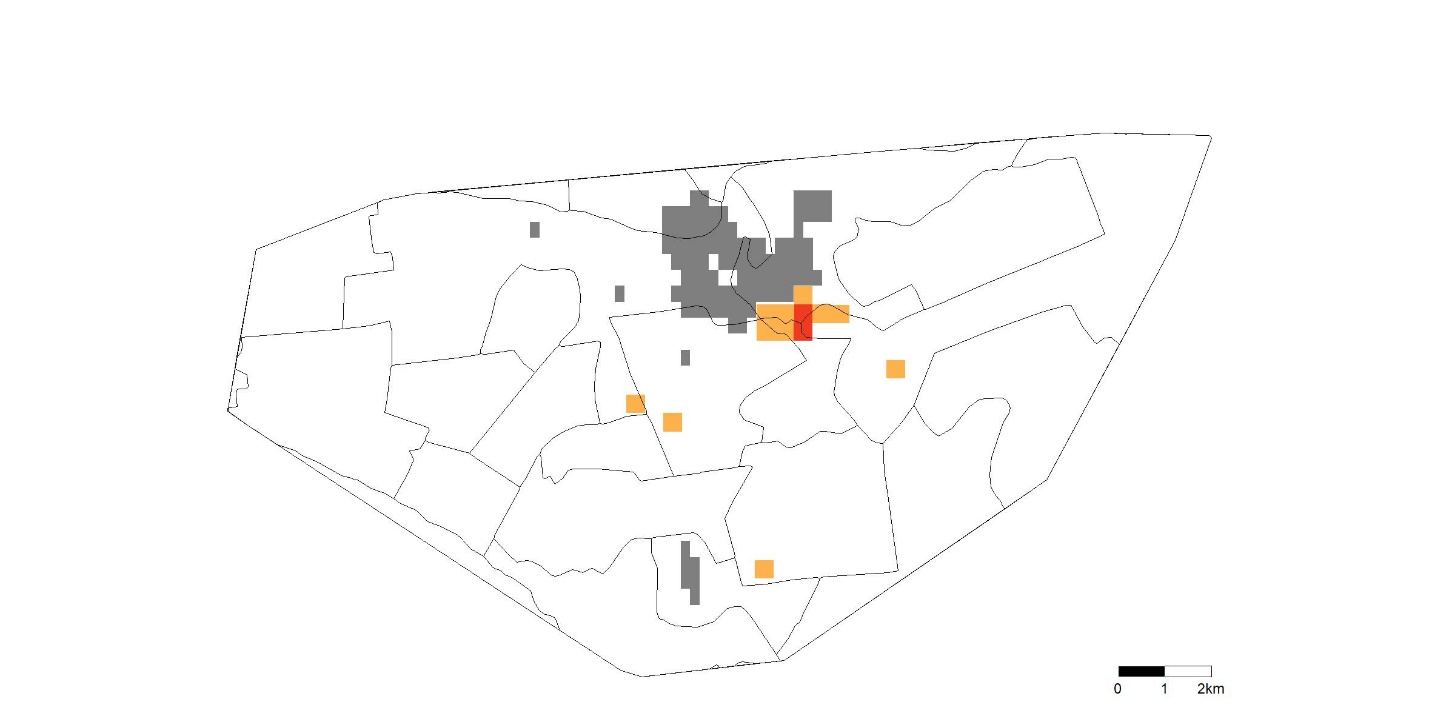


**e)**


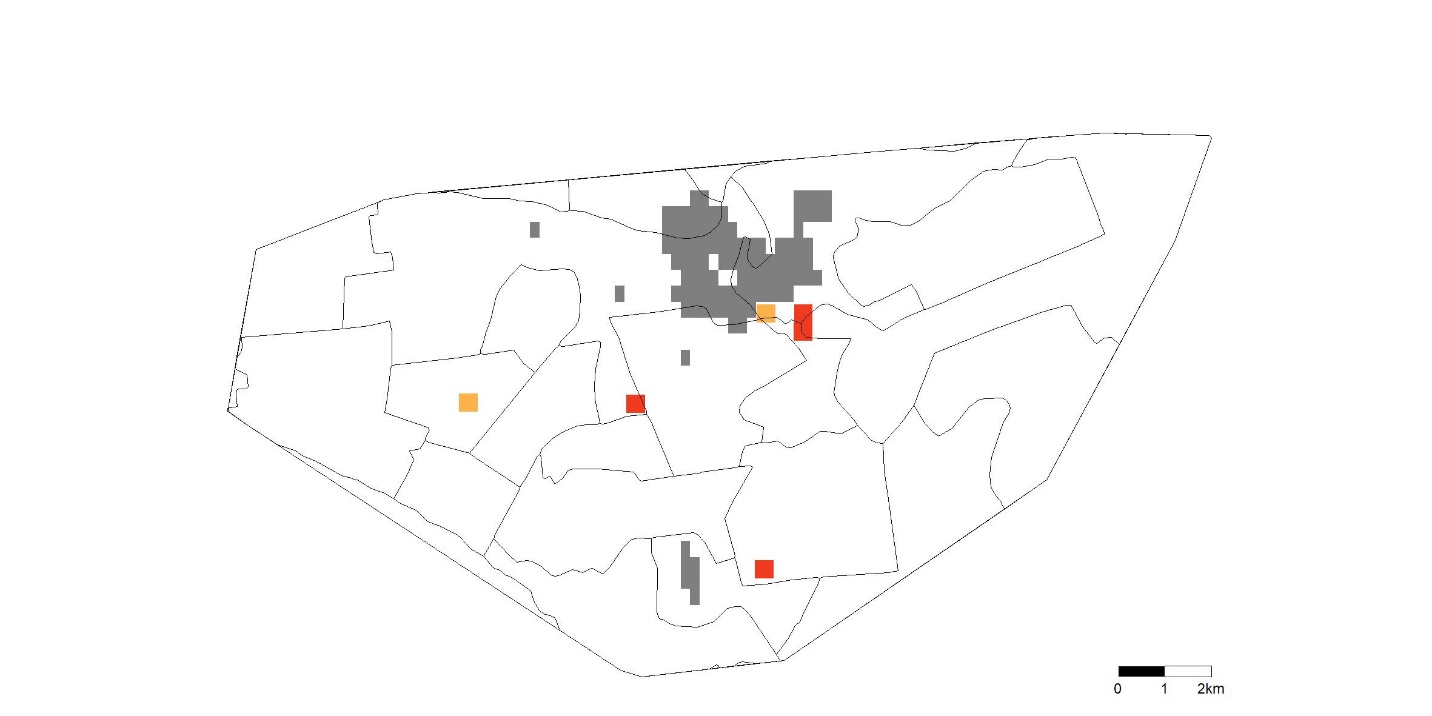


**Figure S2: Sensitivity analysis to grid size.** Model fit using age cut-offs of children as <5 years and adults as >15 years old on different size grids as follows: **(a)** 200 m x 200 m (presented in the main text); **(b)** 300 m x 300 m; **(c)** 400 m x 400 m; **(d)** 500 m x 500 m; (**e)** 600 m x 600 m; **(f)** 700 m x 700 m; **(g)** 800 m x 800 m; **(h)** 900 m x 900 m; **(i)** 1000 m x 1000 m. We highlight those grid cells in which >90% of the modeled posterior distribution of the random effect is above zero (orange), which includes those grid cells in which >95% of the modeled posterior distribution is above zero (red).

**a)**


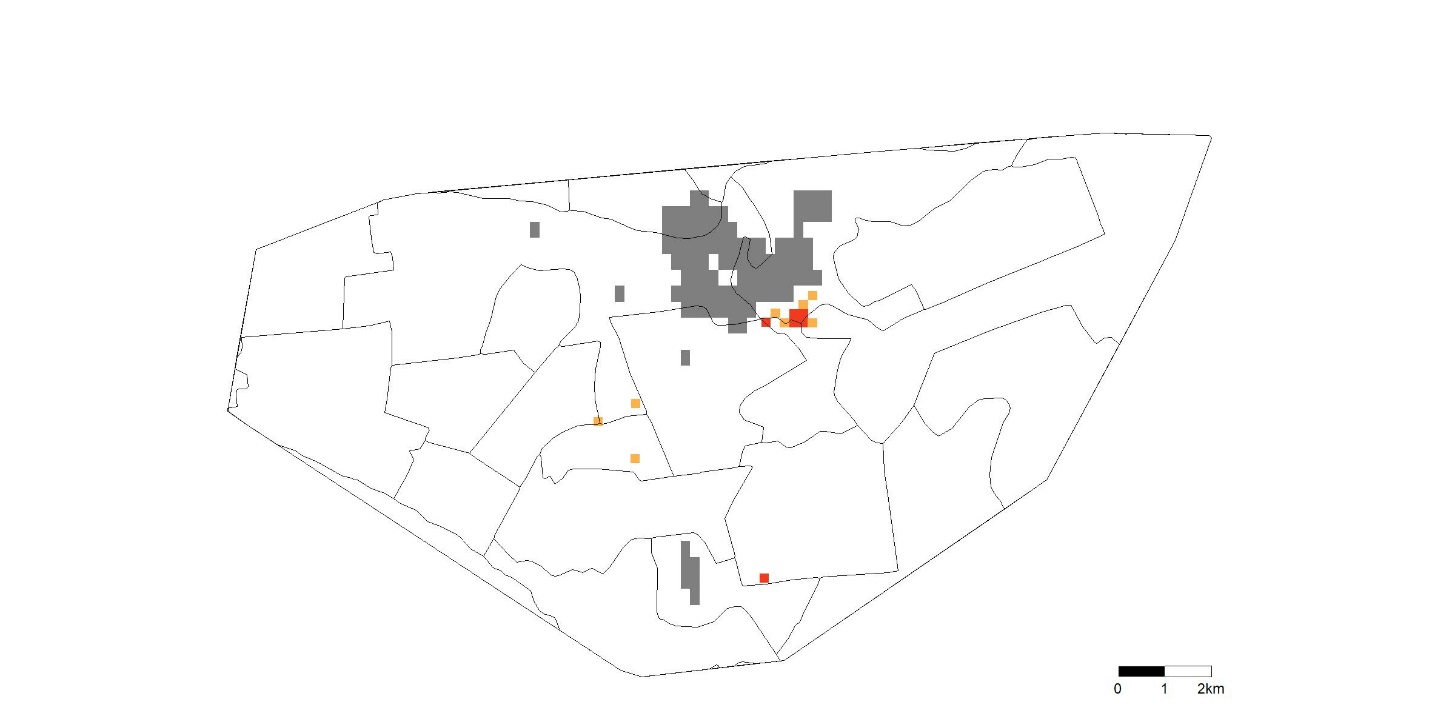


**b)**


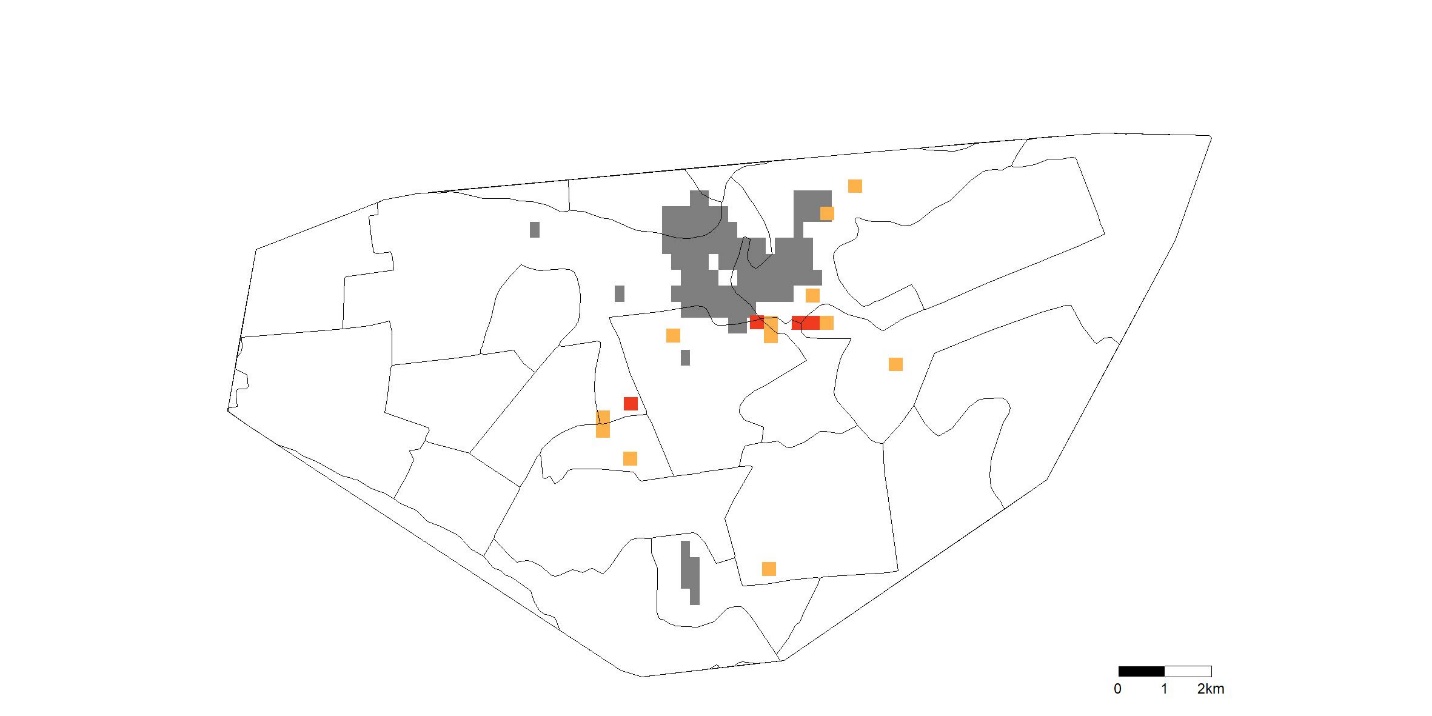


**c)**


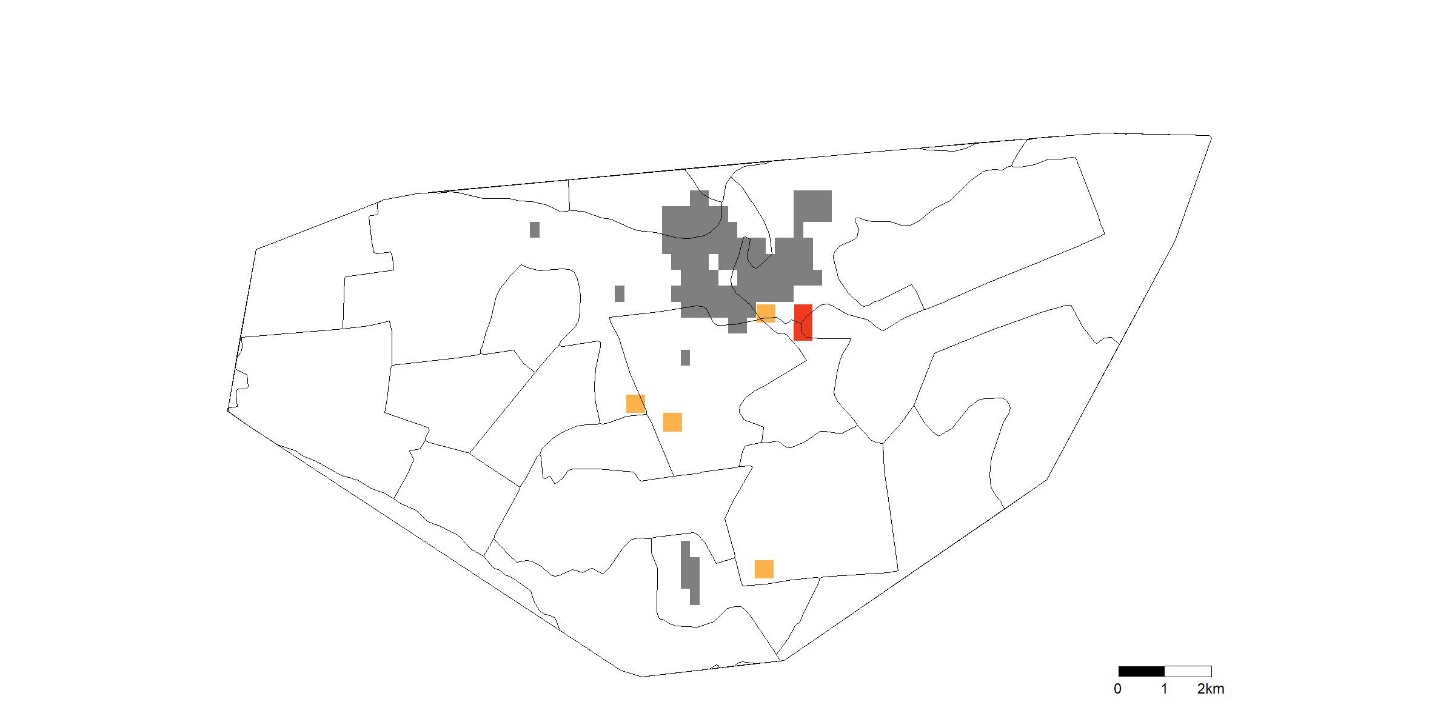


**d)**


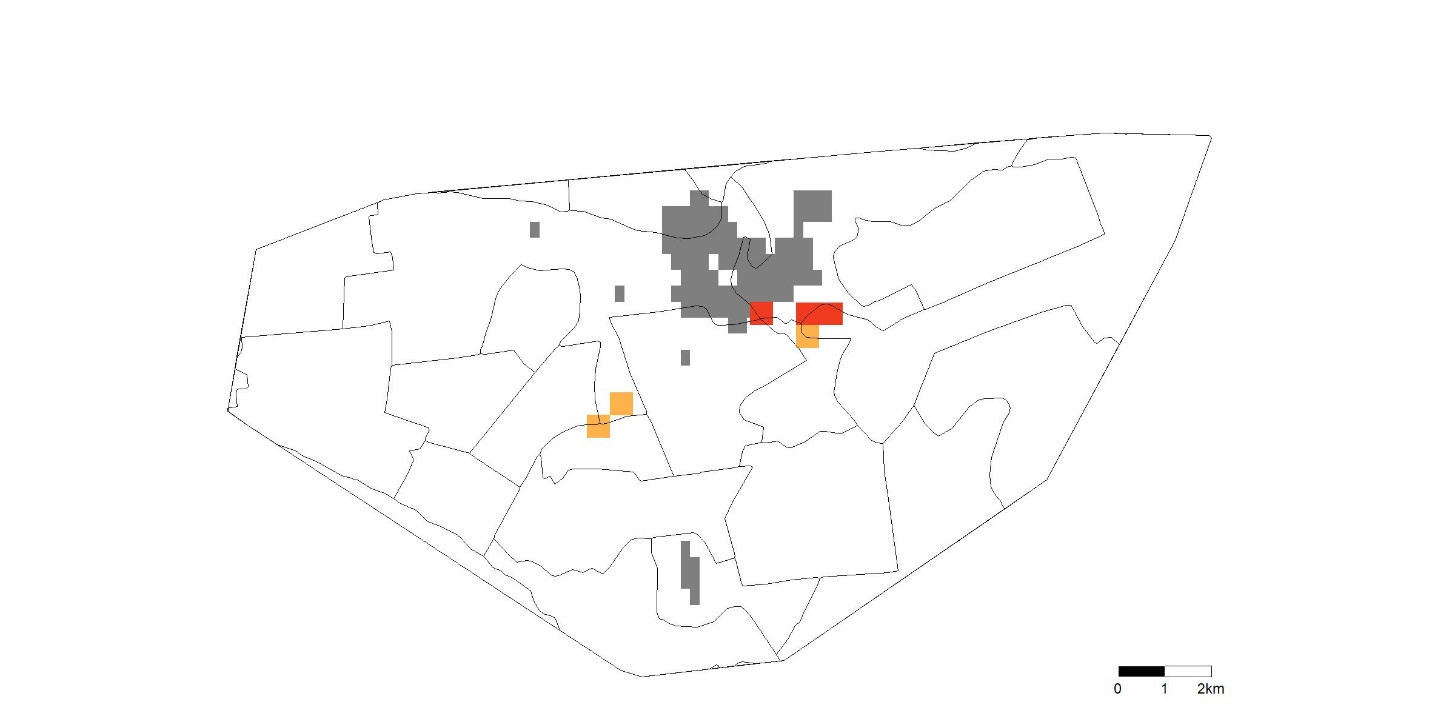


**e)**


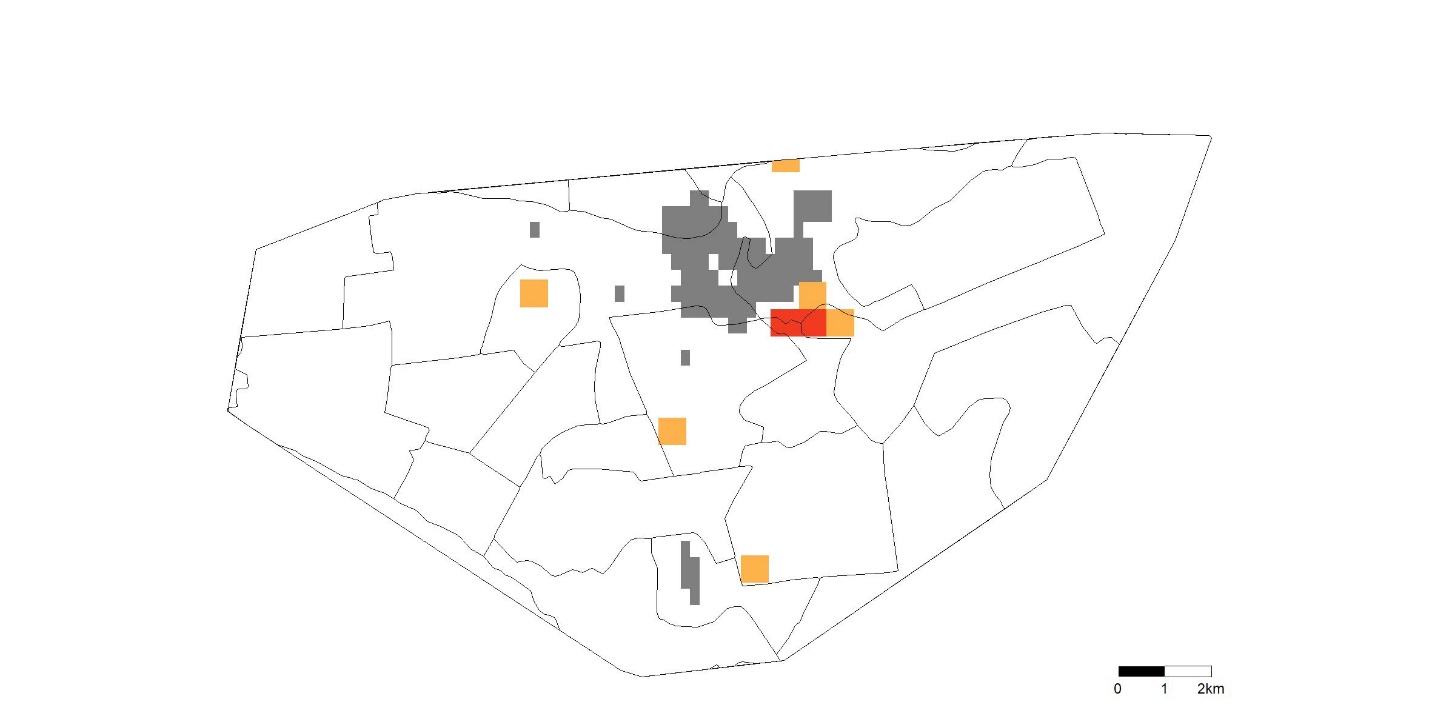


**f)**


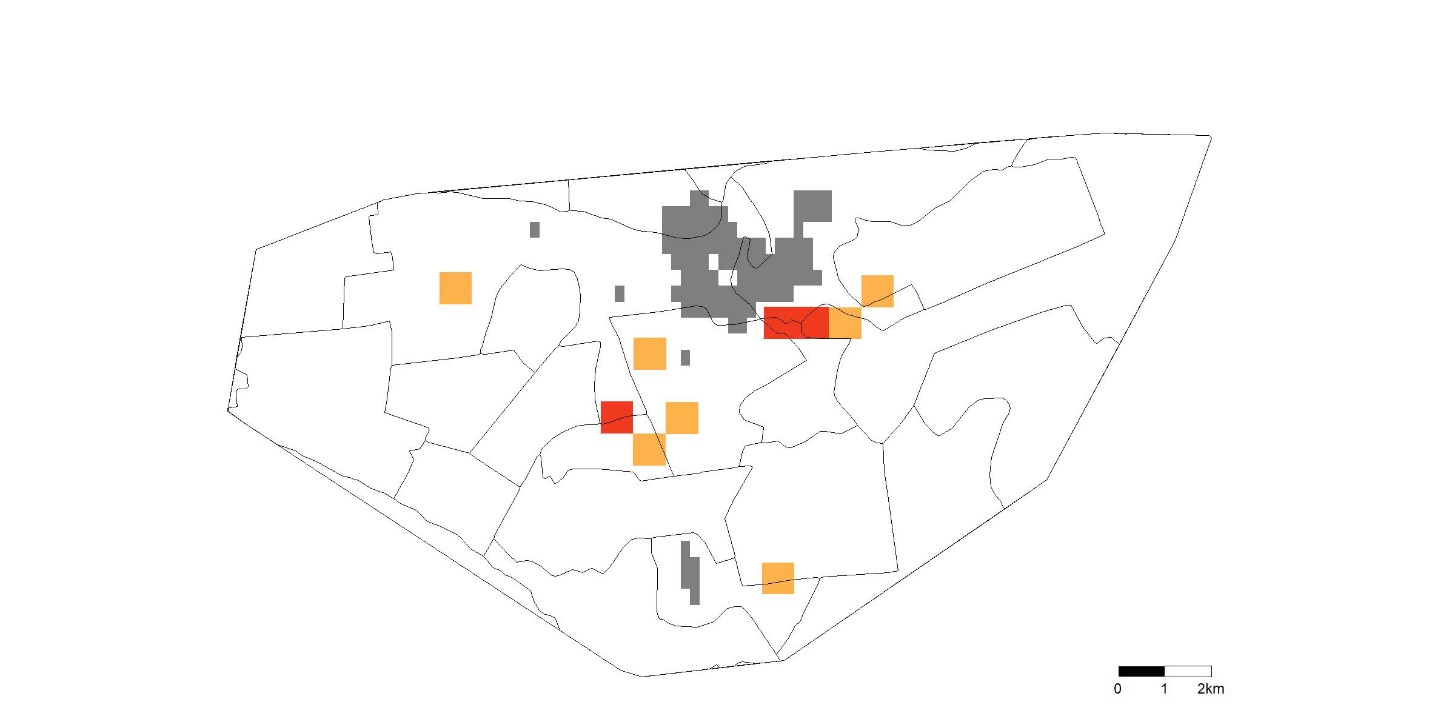


**g)**


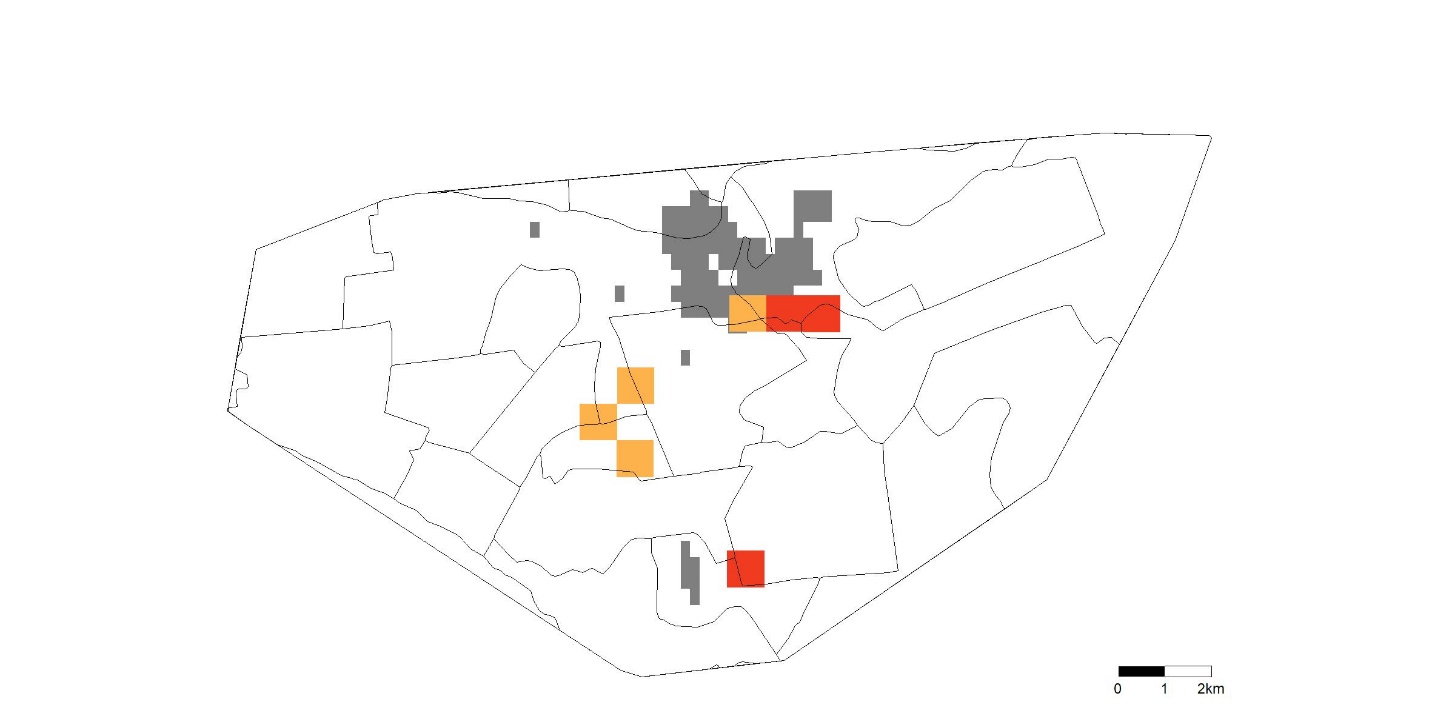


**h)**


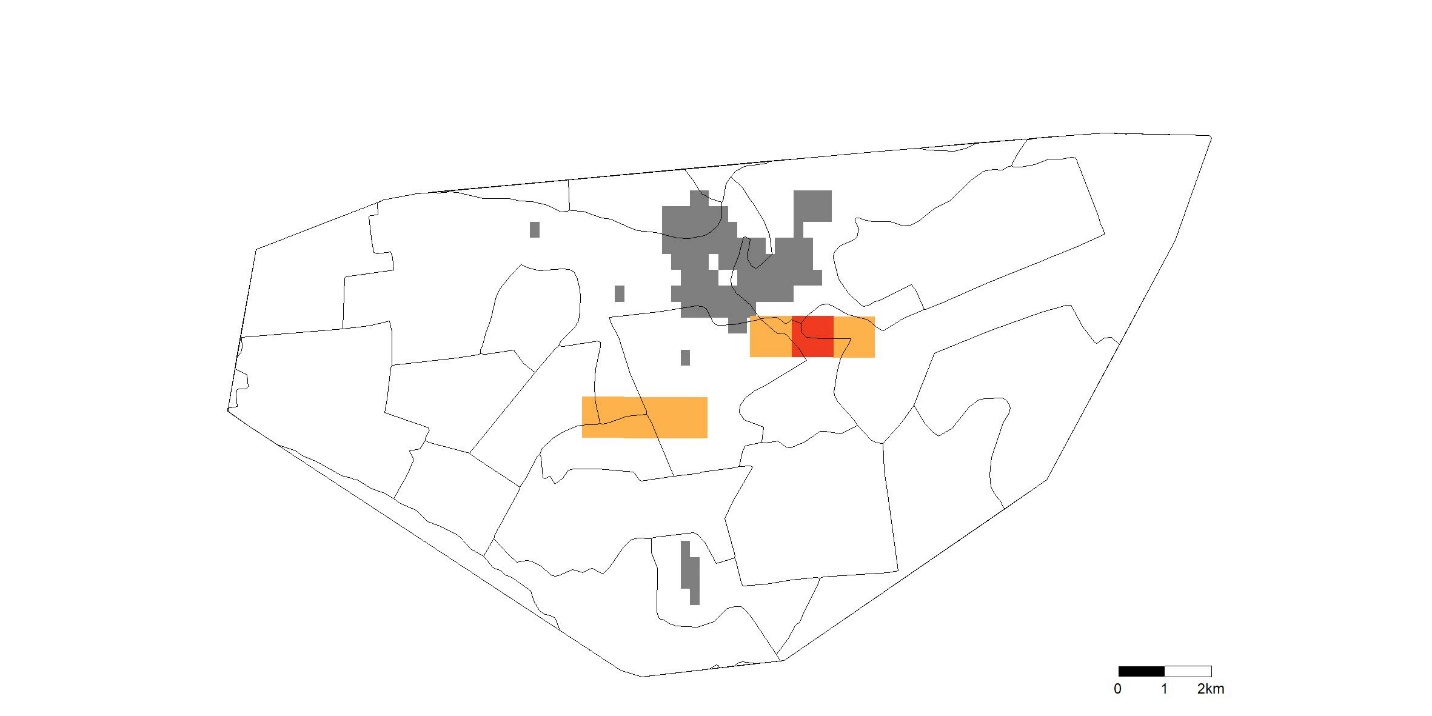


**i)**


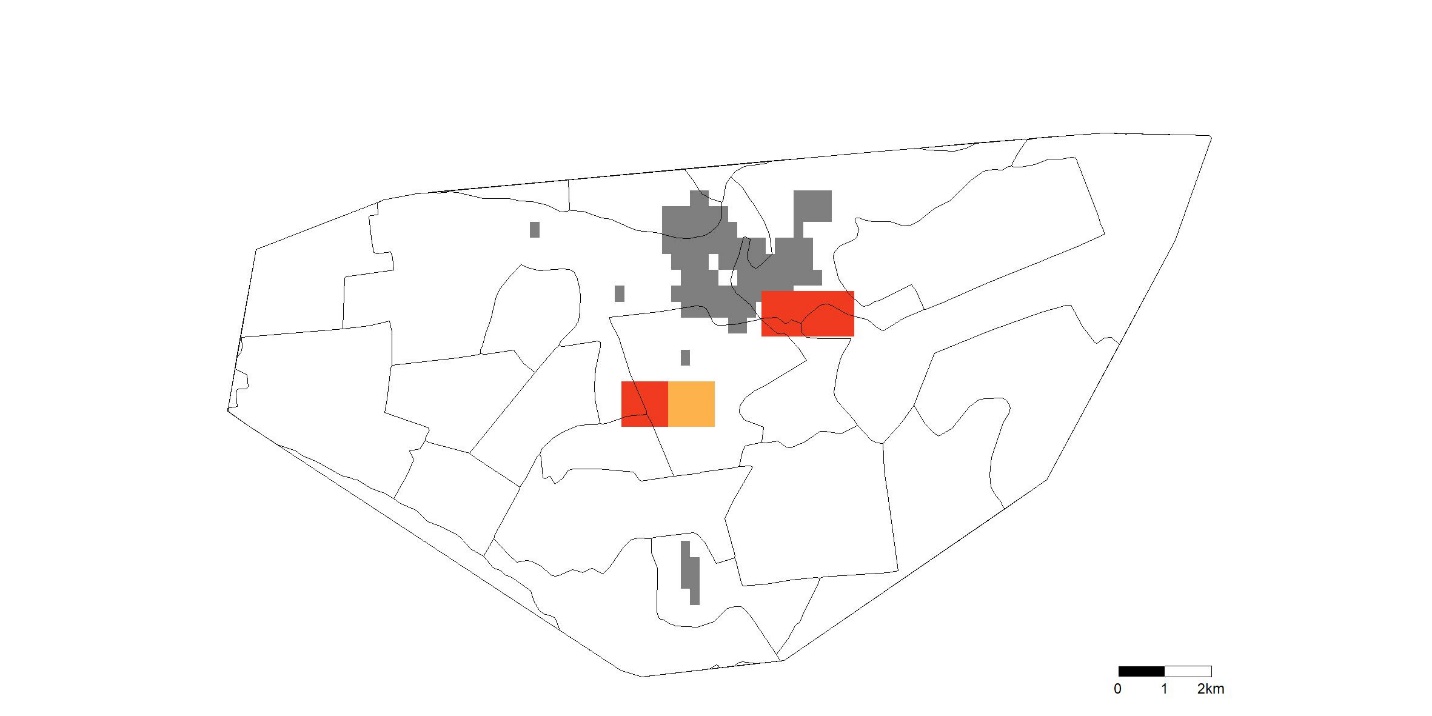

Supplement: Supplementary file 1 — Additional file 1 : Supplementary Information, Table S1, Figure S1-S2. Supplementary Information – Log-Gaussian Cox process details and hierarchical Bayesian spatial model details. Table S1- Hierarchical Bayesian spatial model posterior parameter estimates. Fig S1 – Sensitivity analysis to child and adult age cut-offs. Fig S2 – Sensitivity analysis to grid size. [file 12916_2020_1702_MOESM1_ESM.docx]
